# Determinants of gout therapy non-adherence in patients attending an Egyptian rheumatology outpatient clinic

**DOI:** 10.1186/s12912-026-05073-1

**Published:** 2026-08-28

**Authors:** Samira Saad Ali, Samah Ramadan Shaheen Ibrahim, Manal Saeed Mohammed

**Affiliations:** 1 Medical-Surgical Nursing Department, Faculty of Nursing, Alexandria University, Alexandria, Egypt; 2 Nursing Department College of Applied Medical Sciences, Prince Sattam Bin Abdulaziz University, Wadi Addawasir, Saudi Arabia

**Keywords:** Gout, Medication adherence, Patient compliance, Barrier, Urate-lowering therapy, Egypt, Outpatient clinics

## Abstract

**Background:**

Non-adherence to urate-lowering therapy (ULT) is a significant issue in the management of gout, a prevalent form of inflammatory arthritis that adversely affects patients’ quality of life and burdens healthcare systems. Although ULT is effective in reducing serum uric acid levels, many patients fail to adhere to their medication regimens. Factors contributing to this non-adherence include treatment complexity, medication costs, side effects, forgetfulness, and individual health beliefs, as well as issues related to healthcare providers such as communication and education. Understanding these barriers is crucial for developing effective nursing interventions that enhance adherence and improve patient outcomes.

**Aim:**

Identify and analyze the factors influencing patient adherence to gout therapy and explore the barriers experienced by patients in a clinical setting.

**Methods:**

A descriptive research design was utilized with a convenience sample of 150 adults aged 21–60 years diagnosed with gout, who had been prescribed urate-lowering therapy for at least three months. Data collection occurred from November 1, 2024, to March 30, 2025. Participants were required to have the capacity to provide informed consent and understand the study requirements. Individuals with comorbidities affecting medication adherence, those currently enrolled in other clinical trials, and individuals unable to provide informed consent were excluded from the study. The study employed two questionnaires: a sociodemographic and clinical data questionnaire to gather relevant participant information and Intentional Non-Adherence Scale (INAS) to assess medication adherence. Results: Approximately 60.7% of respondents indicate a low adherence level to medications, while 39.3% report a high adherence level to medications. Key factors affecting adherence include socio-demographic variables, such as higher rates among males and those aged 30–39, as well as sufficient income. In addition, the findings suggest that clinical data correlate with better adherence.

**Conclusion and implications:**

This study highlights critical socio-demographic and clinical characteristics of gout patients, emphasizing the need for enhanced public awareness campaigns, early screening programs, and targeted interventions to improve healthcare access and medication adherence among underserved populations. By addressing barriers such as medication costs and psychological factors through tailored nursing interventions and multidisciplinary collaboration, we can significantly enhance patient outcomes and quality of life for individuals living with gout.

## Introduction

Non-adherence to prescribed medications, particularly for chronic diseases, can have dire consequences. Many patients fail to gain the full benefits of their pharmacotherapy, which can lead to disease progression, disabilities, or even death. The reasons for this non-adherence may stem from factors related to the patient, the treatment itself, or the healthcare team [1]. Most of the population is affected by chronic diseases, with approximately 2.1 billion individuals having chronic condition (World Health Organization, 2023), with projections indicating a more than 70% increase by 2050 [2]. Gouty arthritis is a one of chronic disease and first documented by the ancient Egyptians, and in the fifth century BC, Hippocrates recognized the condition, referring to it as “the un-walkable disease.” This affliction has also been termed the “disease of kings,” highlighting its association with the consumption of specific foods and beverages that are typically accessible to wealthier individuals [3].

Gout is the most common inflammatory arthritis affecting adult worldwide, Gout’s main clinical presentation is in the form of recurrent acute inflammatory arthritis triggered by hyperuricemia and subsequent accumulation of monosodium urate crystal deposition in the joint fluid, cartilage, bones, bursae, tendons, and other sites [4]. In Egypt, the prevalence of gout is estimated to range between 1% and 4% of the general population. Despite the availability of effective urate-lowering therapies, the rate of medication non-adherence is significantly high, with studies suggesting that over 60% of patients do not strictly follow their prescribed treatment regimens. This high rate of non-adherence is primarily driven by socioeconomic barriers, particularly the high cost of medication and frequent shortages in pharmacies. Additional factors contributing to poor compliance include a lack of patient awareness regarding the chronic nature of the disease, the common misconception that treatment is only necessary during painful flares, and forgetfulness due to complex multi-drug regimens for associated comorbidities like hypertension and diabetes [5, 6].

Gout is diagnosed not only by detecting monosodium urate crystals in synovial fluid but also through various imaging techniques, including X-rays, ultrasound, CT, dual-energy CT (DECT), and MRI. These imaging methods aid in predicting patient outcomes and monitoring the condition. According to the 2023 EULAR recommendations, DECT and ultrasound are particularly valuable for assessing gout, as they can track inflammation and crystal deposition. Ultrasound has shown effectiveness in detecting urate deposits around tendons, especially in the early stages of the disease. Recent research has also explored potential diagnostic biomarkers, such as soluble blood markers (e.g., E-cadherin), microRNAs, chemokines (e.g., IP10, IL-8), and growth factors (VEGF-A) in serum and synovial fluid using high-throughput proteomics [7, 8].

Effective pharmacological interventions are essential in the management of gout, which should be approached with a comprehensive treatment plan. The cornerstone of this strategy is urate-lowering therapy (ULT), with allopurinol being the most prescribed medication to lower uric acid levels in patients. For those who are intolerant of allopurinol, alternatives such as febuxostat may be considered. In addition, during acute gout attacks, non-steroidal anti-inflammatory drugs (NSAIDs) and colchicine are frequently used to relieve pain and reduce inflammation. This multifaceted approach ensures that patients receive optimal care tailored to their specific needs [9, 10].

In Egypt, the issuance of the Universal Health Insurance Law (UHI) aimed to achieve Universal Health Coverage (UHC) and provide necessary health services without financial barriers. However, challenges such as medication adherence continue to affect the management of chronic diseases like gout [11]. A recent study revealed that up to 89% of hospitalizations for gout could have been prevented due to insufficient care (Sharma et al., 2014) [12]. This finding is further supported by Tsiamalou et al. (2023) [13], who argued that suboptimal outpatient management and the lack of personalized education are primary drivers for these avoidable admissions. While most patients meet the criteria for Urate-Lowering Therapy (ULT) according to rheumatology guidelines, only a small fraction receive treatment, often at inadequate fixed doses, resulting in a low rate of remission and an increasing disease burden [14].

Despite the availability of effective pharmacological interventions, including urate-lowering therapy (ULT), non-adherence medication remains a significant challenge that adversely affects patient outcomes. Existing literature has extensively explored medication adherence in various chronic conditions; however, the unique aspects of gout such as its episodic nature and the specific treatment protocols have not been adequately addressed. This study aims to investigate the patterns and determinants of medication adherence among individuals with gout, particularly within the Egyptian context, where cultural and socioeconomic factors may influence treatment compliance. By identifying the barriers to adherence and providing insights into patient behaviors, this research seeks to contribute to the optimization of gout management strategies, ultimately improving health outcomes for affected individuals [15, 16].

In Egypt, where gout prevalence affects both genders, non-adherence to prescribed treatments remains a significant public health issue. This adherence gap frequently leads to disease progression, severe complications, and substantial deterioration in patients’ quality of life. The resulting clinical and economic burdens affect both individuals through reduced productivity and increased healthcare costs and the healthcare system at large. Consequently, investigating the root causes of treatment non-adherence represents an essential step toward mitigating disease complications and alleviating the associated socioeconomic impacts on both patients and the national healthcare infrastructure. This study aims to systematically identify and analyze the factors influencing patient adherence to gout therapy, with a particular emphasis on the barriers experienced by patients in clinical settings. Specifically, we seek to address the research question: What are the most prevalent and impactful patient-reported barriers to medication adherence among individuals with gout receiving urate-lowering therapy (ULT)? By exploring these barriers, this research endeavors to contribute to the understanding of the complexities surrounding medication adherence in gout management, ultimately providing evidence-based insights that can inform clinical practices, guide multidisciplinary healthcare teams, and enhance patient outcomes [17].

### Significance of the study

Initially, research on medication adherence focused on unintentional elements, such as forgetfulness or uncertainty regarding treatment plans. Numerous interventions, such as various apps, have focused on providing reminders; however, given that a large portion of nonadherence is deliberate, these methods frequently do not enhance adherence effectively. Intentional nonadherence refers to patients deliberately deciding not to take their medication due to beliefs about their condition or therapy. At present, there is insufficient research investigating the beliefs and perceptions that lead to nonadherence to urate-lowering therapy (ULT) among individuals with gout, with the common assumption that side effects are the primary reason for nonadherence. Patients are frequently asked about side effects, and clinicians typically address these concerns to improve adherence, yet many additional factors may also contribute. A deeper understanding of the reasons for deliberate non-adherence to ULT could assist in creating focused nursing strategies aimed at altering these beliefs and improving adherence behavior [18, 19].

### Theoretical framework for study

The study on patient adherence to gout therapy is guided by the Health Belief Model (HBM) and the Social Determinants of Health (SDH) framework. HBM emphasizes individual perceptions, focusing on perceived severity and susceptibility of gout, perceived benefits of medication, and perceived barriers such as side effects and costs, which influence adherence behaviors. The SDH framework highlights the impact of socio-economic factors, including age, gender, income, education, and employment status, on health outcomes and medication adherence. By integrating these frameworks, the study effectively captures the complex interplay between individual beliefs and socio-demographic factors, guiding the selection of variables related to clinical severity and psychological factors that shape adherence patterns. This comprehensive approach aids in identifying critical areas for intervention to improve patient outcomes in gout management [20].

### Aim of the study

This study aimed to identify and analyze the factors influencing patient adherence to gout therapy and explore the barriers experienced by patients in a clinical setting.

### Design and setting

This cross-sectional study was conducted in patients who presented in the outpatient clinic of rheumatology unit, Main University Hospital in Alexandria, Egypt, it was conducted. This hospital offers free public health services to patients across Egypt. The study followed the STROBE guidelines.

### Participants and sampling

A total of 150 adult patients diagnosed with gout were recruited through convenience sampling methods. Inclusion criteria specified that participants must be adults aged 21–60, have a confirmed gout diagnosis, all participants had been prescribed any urate-lowering therapy (ULT) for at least three months prior to the study. This time frame was established to ensure that participants had sufficient exposure to the medication regimen to identify potential barriers to adherence and evaluate their long-term treatment behaviors. This verified by medical records. Participants also needed to demonstrate the capacity to provide informed consent and understand the study requirements, assessed through a brief interview. Exclusion criteria included individuals with comorbidities affecting medication adherence, such as severe mental health disorders, patients currently enrolled in other clinical trials, and those unable to provide informed consent or lacking cognitive capacity.

### Sample size determination

The required sample size was initially calculated using Epi Info 7.2, which indicated a need for approximately 384 participants based on a predicted frequency of 50%, a 95% confidence level, and a 5% margin of error. After applying the finite population correction for the total population of 180 patients with gout, the adjusted sample size was determined to be 150. This sample size was deemed adequate for robust statistical analyses, with an anticipated effect size (f²) of 0.15, reflecting a moderate effect, and a targeted statistical power of 0.80 (80%) to minimize the risk of Type II errors. Consequently, a convenience sample of 150 patients with gout was selected to ensure adequate representation.

### Study instruments

The following instruments were used to collect data on barriers to medication adherence experienced by patients with gout in clinical practice:

Two tools were used to collect the necessary data about the study variables.

**Tool I: Patients’ socio-demographic characteristics and clinical data assessment sheet**; it was established by the researcher and included age, gender, marital status. residence, level of education, occupation, income, comorbidities, treatment history, previously diagnosed with gout and family history, to identify risk factors for non-adherence.

**Tool (II): Intentional Non-Adherence Scale (INAS).**

The (INAS) developed by (Weinman et al., 2018) to assess intentional nonadherence to any prescribed medications and the reasons behind this behavior. The scoring system for the INAS is based on a 5-point Likert scale, where participants rate their agreement with 22 items ranging from 1 (strongly disagree) to 5 (strongly agree). The total score is calculated by summing the scores of all 22 items, resulting in a range from 22 to 110. To facilitate interpretation, the total score is then divided by the maximum possible score (110) and multiplied by 100 to obtain a percentage. Higher scores indicate a greater level of intentional nonadherence. Based on the median score of the participants, individuals were categorized into two groups: those with a high level of intentional nonadherence (≥ median total score) and those with a low level (< median total score).

The instrument demonstrated strong reliability and validity. The internal consistency, measured by Cronbach’s alpha, was 0.85, indicating excellent reliability. Additionally, content validity was ensured through expert review during the development of the scale, and construct validity was supported by factor analysis, which confirmed that the items grouped into the expected subscales: Resisting Illness, Testing Treatment, and Drug-Specific Concerns. These findings indicate that the INAS is a reliable and valid tool for assessing intentional nonadherence to medication among patients with gout, with a clear ability to differentiate between levels of adherence based on the percentage scores [19, 21, 22].

### Procedure

#### Pilot study

The study tools including tool I, II and were carefully translated into Arabic by bilingual experts proficient in both English and Arabic, ensuring accuracy and cultural relevance. The face and content validity of the translated tools were tested by an expert panel specializing in medical-surgical nursing. A pilot study involving 10% (5 participants) of patients with gout, was conducted to assess the clarity and applicability of tools and they did not participate in the actual study. Cronbach’s alpha test was utilized to examine the reliability of the study tools. Subsequently, Intentional Non-Adherence Scale (INAS) yielded significant results on the reliability test (0.88).

#### Data collection

Data collection for the study occurred over three months, beginning in November 2024. Eligible patients were identified through medical records and physician referrals. The researcher-initiated contact by introducing himself, explaining the study’s objectives, and assuring participants that their responses would remain confidential. The data collection method involved structured face-to-face interviews, during which researcher-administered surveys were utilized. Participants responded to questions presented in Tools 1 and 2. After obtaining informed consent, the interviews were conducted in a private, quiet, and well-ventilated room within the rheumatology unit. Throughout the process, participants received consistent instructions, and the researcher was available to promptly address any questions or concerns raised.

Each data collection session lasted approximately 20 min, during which researchers ensured that all responses were complete and accurately recorded. A detailed consent form was provided to each participant, outlining the study’s objectives, the voluntary nature of participation, and the right to withdraw at any time without any impact on their medical care. Participants were encouraged to ask questions and discuss the study with family or friends before making their decision. Informed consent was obtained only after participants fully understood the information provided and voluntarily agreed to participate in the study.

### Statistical analysis of data

Data were analyzed using IBM SPSS Statistics (Version 25), with initial data cleaning performed through frequency analysis, cross-tabulation, and manual review to ensure dataset integrity. Descriptive statistics, including means, standard deviations, and frequencies, were utilized to characterize the sociodemographic and clinical profiles of the participants, while Chi-square tests were employed to evaluate associations between categorical variables. To identify predictors of medication adherence measured by the Intentional Non-Adherence Scale (INAS), a multivariable linear regression analysis was conducted. This model controlled for potential confounding by including demographic and clinical covariates, with model fit evaluated via the coefficient of determination (R2 = 0.466) and adjusted R2 (0.419). Statistical significance was established of *p* ≤ 0.05 A significance level of for all analyses.

## Results

A total of **175** patients were initially contacted for participation in the study, of whom **150** met the inclusion criteria and completed the assessment, resulting in a high response rate of **85.7%**. All participants (*n* = 150) had been prescribed urate-lowering therapy (ULT) for at least three months prior to the study, ensuring they had sufficient experience with the treatment regimen to identify adherence barriers. While the study tool focused on general adherence behaviors and did not record specific medication names, the sample provided a comprehensive basis for analyzing clinical challenges and patient perceptions within the nursing context.

Table 1 shows the socio-demographic characteristics of the study population reveal a predominance of males 60% over females 40%. The age distribution indicates a significant number of participants are aged 40–49 years 38.7%, while a notable proportion is over 60 years 10.0%. Education levels show that most participants have basic education 43.3%, 26.0% Cleric work and the majority are married 45.3% in addition to 50.7% living in urban areas, 93.3% with insufficient income and 80.0% treated in university hospital.

**Table 1:** Socio-demographic data (*n* = 150)

Socio-demographic data	No.	%
**Sex**		
Male	90	60.0
Female	60	40.0
**Age (years)**		
20:29	4	2.7
30:39	51	34.0
40:49	58	38.7
50:59	22	14.7
> 60	15	10.0
**Mean ± SD.**	**44.46 ± 10.32**	
**Education**		
Illiterate	5	3.3
Read and write	25	16.7
Basic education	65	43.3
Secondary	31	20.7
University	24	16.0
**Occupation**		
Manual	31	20.7
Cleric work	39	26.0
House wife	43	28.7
Not work	19	12.7
Retirement	18	12.0
**Marital status**		
Single	22	14.7
Married	68	45.3
Divorced	40	26.7
Widow	20	13.3
**Residence**		
Urban	76	50.7
Rural	74	49.3
**Income**		
Enough	10	6.7
Not enough	140	93.3
**Treatment**		
Health insurance	16	10.7
Private	14	9.3
University hospital	120	80.0

Table 2 reveals the clinical data highlights that a significant number of participants have associated diseases with diabetes mellitus reported by 33.3% and hypertension by 26.0%. The data also shows that over half of the participants, 51.3%, take prescribed medications. Awareness of gout primarily occurred accidentally during a follow -up with the doctor 51.3% or during severe symptoms 48.7%. The duration of illness reveals that 28.0% have had gout for less than three months, while 51.3% have had it for over six months. Additionally, 38.0% have a history of hospitalization, Lastly, 16.7% reported a family history of gout.

**Table 2:** Clinical data (*n* = 150)

Clinical data	No.	%
**9- The presence of Associated Disease**		
Hypertension	39	26.0
Heart. Disease	4	2.7
D.M	50	33.3
Liver disease	0	0
Kidney disease	8	5.3
Blood disorders	0	0
Endocrine disorders	20	13.3
**10-Do you take Prescribed Medications**		
Yes	77	51.3
No	73	48.7
**11-How did you know have Gout**		
At onset of symptom	77	51.3
When symptoms become severe	73	48.7
Accidentally during a follow -up with the doctor	77	51.3
**12-Duration of illness**		
< 3 months	42	28.0
3:6 months	31	20.7
> 6 months	77	51.3
**Past history**		
**13-Did you have Previous Hospitalization**		
Yes	57	38.0
No	93	62.0
**14- Did you have Previous Surgery**		
Yes	50	33.3
No	100	66.7
**15-Do you have Family History of Gout**		
Yes	25	16.7
No	125	83.3

Table 3 presents responses to barriers for medication adherence indicate that a substantial proportion of participants are not convinced about the necessity or effectiveness of their medications. The data indicates that a significant proportion of respondents expressed doubts about the necessity of their medications, with 29.3% agreeing that they need to see if their illness is still present before continuing treatment, highlighting a potential lack of understanding of their condition’s chronic nature. 34.0% agree with this medication is not right for their condition. Concerns about the side effects of medication are prominent, with 77.3% expressing discontent with side effects and similar sentiments regarding chemicals in the body. This suggests that side effects play a significant role in patients’ decisions to adhere to medication regimens.

**Table 3:** Tool (II): intentional non-adherence scale (INAS). (*n* = 150)

	Strongly disagree		Disagree		Neutral		Agree		Strongly agree	
	No.	%	No.	%	No.	%	No.	%	No.	%
1-To see if my illness is still there	33	22.0	59	39.3	10	6.7	44	29.3	4	2.7
2-To see if I can without it	11	7.3	43	28.7	18	12.0	56	37.3	22	14.7
3- To see if I really need it	0	0.0	6	4.0	62	41.3	52	34.7	30	20.0
4-Because I am not convinced that the medicine is really right for me	0	0.0	45	30.0	54	36.0	51	34.0	0	0.0
5- Because I am not sure that the doctor chose the right medicine for me	0	0.0	45	30.0	40	26.7	59	39.3	6	4.0
6-To give my body a rest from the medicine	0	0.0	22	14.7	12	8.0	107	71.3	9	6.0
7-Because the medicine is harsh on my body	4	2.7	21	14.0	14	9.3	102	68.0	9	6.0
8-Because I don`t like the medicine to accumulate in my body	0	0.0	15	10.0	40	26.7	61	40.7	34	22.7
9-Because my body is sensitive to the effects of medicine	6	4.0	27	18.0	12	8.0	67	44.7	38	25.3
10-Brcause I don`t like the side effects	0	0.0	0	0.0	27	18.0	116	77.3	7	4.7
11-I don`t like chemicals in my body	0	0.0	0	0.0	29	19.3	109	72.7	12	8.0
12- Because it may affect the body`s own natural healing processes	0	0.0	0	0.0	68	45.3	70	46.7	12	8.0
13- Because I think I am on too high a dose	0	0.0	31	20.7	28	18.7	84	56.0	7	4.7
14- Because I think the drug might become less effective over time	0	0.0	0	0.0	65	43.3	77	51.3	8	5.3
15- Because I worry about becoming dependent on my medicine	0	0.0	28	18.7	0	0.0	107	71.3	15	10.0
16- Because I want to think of myself as healthy person again	0	0.0	7	4.7	0	0.0	110	73.3	33	22.0
17-Because it reminds me that I have an illness	4	2.7	0	0.0	0	0.0	110	73.3	36	24.0
18- Because I want to lead a normal life again	0	0.0	0	0.0	4	2.7	132	88.0	14	9.3
19-Because it is good not to have to remember	2	1.3	0	0.0	9	6.0	128	85.3	11	7.3
20-Because it is inconvenient to take all the time	0	0.0	0	0.0	52	34.7	91	60.7	7	4.7
21-Because the drug schedule doesn`t fit with my lifestyle	0	0.0	41	27.3	26	17.3	80	53.3	3	2.0
22-Because I don`t think the treatment is worth it	40	26.7	60	40.0	13	8.7	34	22.7	3	2.0
**Total score**	**78.25 ± 8.72**									
**Mean percent score**	**63.92 ± 9.91**									
**Level**										
** Low adherence**	**91 (60.7%)**									
** High adherence**	**59 (39.3%)**									

Furthermore, 71.3% of participants agreed that they worry about becoming dependent on their medications, this concern may drive patients to skip doses or discontinue use, which reflects a common psychological barrier to adherence. In relation to the inconvenience of medication schedules and their fit with personal lifestyles significantly impact adherence. Over 60% of respondents indicated that the drug schedule does not align with their lifestyle Additionally, 26.0% expressed skepticism regarding whether the prescribed medicine is appropriate for them, suggesting a disconnect between patients and their healthcare providers that could lead to non-adherence. The responses also indicate that many participants are unsure if their doctor has chosen the right medication, with 29.3% agreeing with this statement. The total score suggests a mean adherence level of 63.92%, indicating a low level of adherence but also highlighting significant barriers that need to be addressed.

Table 4 this table reveals significant relationships between medication adherence and various socio-demographic factors among 150 participants. Males 72.9% demonstrated higher adherence compared to females 27.1%, and adherence varied significantly by age, peaking in the 40–49 age group 54.2%. Education played a crucial role, with only 1.1% of university-educated individuals reporting low adherence. Housewives 42.4% showed better adherence than clerical workers 18.6%, and divorced individuals had higher adherence 39.0% compared to married ones 30.5%. Urban residents 62.7% outperformed rural residents 37.3%, and the adherence rates indicate that among participants with insufficient income 84.7% adhered to treatment, while among those with sufficient income, only 15.3% adhered. These findings underscore the importance of considering socio-demographic factors in developing strategies to improve medication adherence.

**Table 4:** Relation between Adherence and Socio-demographic data (*n* = 150)

Socio-demographic data	Adherence				χ^2^	*p*
	Low (*n* = 91)		High (*n* = 59)			
	No.	%	No.	%		
**Sex**						
Male	47	51.6%	43	72.9%	6.724*	0.010*
Female	44	48.4%	16	27.1%		
**Age (years)**						
20:29	1	1.1%	3	5.1%		
30:39	36	39.6%	15	25.4%		
40:49	26	28.6%	32	54.2%	16.425*	0.002*
50:59	19	20.9%	3	5.1%		
> 60	9	9.9%	6	10.2%		
**Education**						
Illiterate	5	5.5%	0	0.0%		
Read and write	17	18.7%	8	13.6%		
Basic education	47	51.6%	18	30.5%	40.254*	< 0.001*
Secondary	21	23.1%	10	16.9%		
University	1	1.1%	23	39.0%		
**Occupation**						
Manual	18	19.8%	13	22.0%		
Cleric work	28	30.8%	11	18.6%		
House wife	18	19.8%	25	42.4%	11.418*	0.022*
Not work	15	16.5%	4	6.8%		
Retirement	12	13.2%	6	10.2%		
**Marital status**						
Single	13	14.3%	9	15.3%		
Married	50	54.9%	18	30.5%		
Divorced	17	18.7%	23	39.0%	10.539*	0.014*
Widow	11	12.1%	9	15.3%		
**Residence**						
Urban	39	42.9%	37	62.7%	5.645*	0.018*
Rural	52	57.1%	22	37.3%		
**Income**						
Enough	1	1.1%	9	15.3%		
Not enough	90	98.9%	50	84.7%	11.529*	0.001*
**Treatment**						
Health insurance	11	12.1%	5	8.5%		
Private	7	7.7%	7	11.9%	1.107	0.575
University hospital	73	80.2%	47	79.7%		

χ^2^: Chi square test

*: Statistically significant at *p* ≤ 0.05

Table 5 this analysis examines the relationship between medication adherence and clinical data among participants, highlighting significant associations. Notably, adherence was markedly higher among those who took prescribed medications (71.2% in the high adherence group vs. 38.5% in the low group, χ² = 15.344, *p* < 0.001). Additionally, individuals who recognized their gout at the onset of symptoms had better adherence (33.9%) compared to those who only acknowledged it when symptoms worsened (44.1% vs. 74.7%, χ² = 32.943, *p* < 0.001). The duration of illness also influenced adherence, with those suffering for 3–6 months showing low adherence (χ² = 25.548, *p* < 0.001). Previous surgeries were associated with lower adherence rates (20.3% vs. 41.8%, χ² = 7.390, *p* = 0.007). Other clinical factors, such as the presence of associated diseases and family history of gout, did not show significant differences. These findings suggest that medication management, timely diagnosis, and the duration of illness are critical factors affecting adherence.

**Table 5:** Relation between adherence and clinical data (*n* = 150)

Clinical data	Adherence				χ^2^	*p*
	Low (*n* = 91)		High (*n* = 59)			
	No.	%	No.	%		
**The presence of associated disease**						
Hypertension	25	27.5%	14	23.7%	0.261	0.610
Heart. Disease	3	3.3%	1	1.7%	0.354	1.000
D.M	32	35.2%	18	30.5%	0.349	0.555
Liver disease	0	0.0%	0	0.0%	-	-
Kidney disease	4	4.4%	4	6.8%	0.403	0.526
Blood disorders	0	0.0%	0	0.0%	-	-
Endocrine disorders	19	20.9%	1	1.7%	11.400*	0.001*
**Do you take prescribed medications**						
Yes	35	38.5%	42	71.2%	15.344*	< 0.001*
No	56	61.5%	17	28.8%		
**How did you know have gout**						
At onset of symptom	1	1.1%	20	33.9%		
When symptoms become severe	68	74.7%	26	44.1%	32.943*	< 0.001*
Accidentally during a follow -up with the doctor	22	24.2%	13	22.0%		
**Duration of illness**						
< 3 months	20	22.0%	22	37.3%		
3:6 months	31	34.1%	0	0.0%	25.548*	< 0.001*
> 6 months	40	44.0%	37	62.7%		
**Past history**						
**Did you have previous hospitalization**						
Yes	30	33.0%	27	45.8%	2.487	0.115
No	61	67.0%	32	54.2%		
**Did you have previous surgery**						
Yes	38	41.8%	12	20.3%	7.390*	0.007*
No	53	58.2%	47	79.7%		
**Do you have family history of gout**						
Yes	13	14.3%	12	20.3%	0.944	0.331
No	78	85.7%	47	79.7%		

χ^2^: Chi square test

*: Statistically significant at *p* ≤ 0.05

Table 6 reveals the bivariate correlation between patient adherence and their socio-demographic and clinical characteristics (*n* = 150). A statistically significant positive correlation was found between adherence and higher education levels (rs = 0.372, *p* = 0.001). Conversely, significant negative correlations were observed between adherence and female sex (rs = -0.212, *p* = 0.009), rural residence (rs = -0.194, *p* = 0.017), and insufficient income (rs = -0.277, *p* = 0.001). Regarding clinical data, adherence was significantly lower among patients with endocrine disorders (rs = -0.276, *p* = 0.001), those not taking prescribed medications (rs = -0.320, *p* = 0.001), and those diagnosed with gout accidentally (rs = -0.266, *p* = 0.001). Interestingly, a history of no previous surgery showed a significant positive correlation with adherence (rs = 0.222, *p* = 0.006). No other variables, including age, marital status, or duration of illness, reached statistical significance (*p* > 0.05).

**Table 6:** Bivariate correlation between adherence and socio-demographic data (*n* = 150)

	Adherence	
	rs	*P*
Sex (Female)	-0.212*	0.009*
Age (years)	-0.086	0.297
Education (high)	0.372*	0.001*
Occupation (Not working)	-0.014	0.861
Marital status (Married)	0.158	0.053
Residence (Rural)	-0.194*	0.017*
Income (Not enough)	-0.277*	0.001*
Treatment (University hospital)	0.003	0.974
The presence of associated disease		
Hypertension (yes)	-0.042	0.612
Heart. Disease (yes)	-0.049	0.555
D.M (yes)	-0.048	0.558
Kidney disease (yes)	0.052	0.529
Endocrine disorders (yes)	-0.276*	0.001*
Do you take prescribed medications (No)	-0.320*	0.001*
How did you know have gout (Accidentally)	-0.266*	0.001*
Duration of illness	0.056	0.495
Past history		
Did you have previous hospitalization (No)	-0.129	0.116
Did you have previous surgery (No)	0.222*	0.006*
Do you have family history of gout (No)	-0.079	0.334

r_s_: Spearman coefficient

*: Statistically significant at *p* ≤ 0.05

Table 7 presents the multivariate linear regression analysis identifying factors that independently predict intentional non-adherence scale (INAS score percentage) among participants. Age was significantly and negatively associated with the INAS score (B = -2.743, *p* = 0.002), confirming that older age is associated with lower intentional non-adherence scores, and thus, better medication adherence. Conversely, marital status showed a significant positive coefficient (B = 3.427, *p* = 0.001), indicating that married individuals reported higher levels of intentional non-adherence compared to unmarried participants. Socioeconomically, reporting an insufficient income was the strongest negative predictor of the INAS score (B = -9.216, *p* < 0.001), meaning participants with limited financial resources demonstrated lower intentional meaning participants with limited financial resources demonstrated lower intentional non-adherence (better adherence). Clinically, not taking other prescribed medications (B = -4.228, *p* = 0.004), discovering gout accidentally during a routine doctor follow-up (B = -2.074, *p* = 0.043), and having a longer duration of illness exceeding 6 months (B = -1.709, *p* = 0.040) were all significantly associated with lower INAS scores, reflecting lower non-adherence (better adherence) profiles within these specific patient groups.

**Table 7:** Multivariate analysis linear regression for factors affecting adherence (*n* = 150)

Factors	B	Beta	t	*p*	95% CI	
					LL	UL
**Sex (female)**	-2.895	-0.163	-1.802	0.074	-6.071	0.281
**Age (years)**	-2.743	-0.314	-3.110*	0.002*	-4.487	-0.999
**Education (high)**	1.290	0.153	1.833	0.069	-0.102	2.682
**Occupation (**Not work**)**	0.797	0.116	1.260	0.210	-0.454	2.047
**Marital status (married)**	3.427	0.352	3.315*	0.001*	1.383	5.471
**Residence (**Rural**)**	1.286	0.074	0.984	0.327	-1.298	3.869
**Income (**Not enough**)**	-9.216	-0.265	-3.758*	< 0.001*	-14.065	-4.367
**Endocrine disorders (yes)**	-2.435	-0.095	-1.267	0.207	-6.235	1.365
**Do you take prescribed medications (**No**)**	-4.228	-0.243	-2.913*	0.004*	-7.098	-1.358
**How did you know have gout (**Accidentally during a follow -up with the doctor**)**	-2.074	-0.144	-2.038*	0.043*	-4.087	-0.062
**Duration of illness (**> 6 months**)**	-1.709	-0.169	-2.071*	0.040*	-3.340	-0.077
**Do you have previous surgery (**No**)**	-0.154	-0.008	-0.110	0.913	-2.938	2.630
**R**^**2**^**= 0.466**,** adjusted R**^**2**^ **= 0.419**, F = 9.964*, *p* < 0.001*						

F, p: f and p values for the model R^2^: Coefficient of determination; B: Unstandardized Coefficients Beta: Standardized Coefficients; t: t-test of significance CI: Confidence interval; LL: Lower limit UL: Upper Limit

*: Statistically significant at *p* ≤ 0.05

**Statistical analysis of the data**

Data were fed to the computer and analyzed using IBM SPSS software package version 26.0. Qualitative data were described using number and percent. Quantitative data were described using mean, standard deviation, median. Significance of the obtained results was judged at the 5% level

**The used tests were**

**1 - Chi-square test**

For categorical variables, to compare between different groups

**2 – Regression**

To detect the most independent factor affecting **Adherence**

**Fig. 1:**
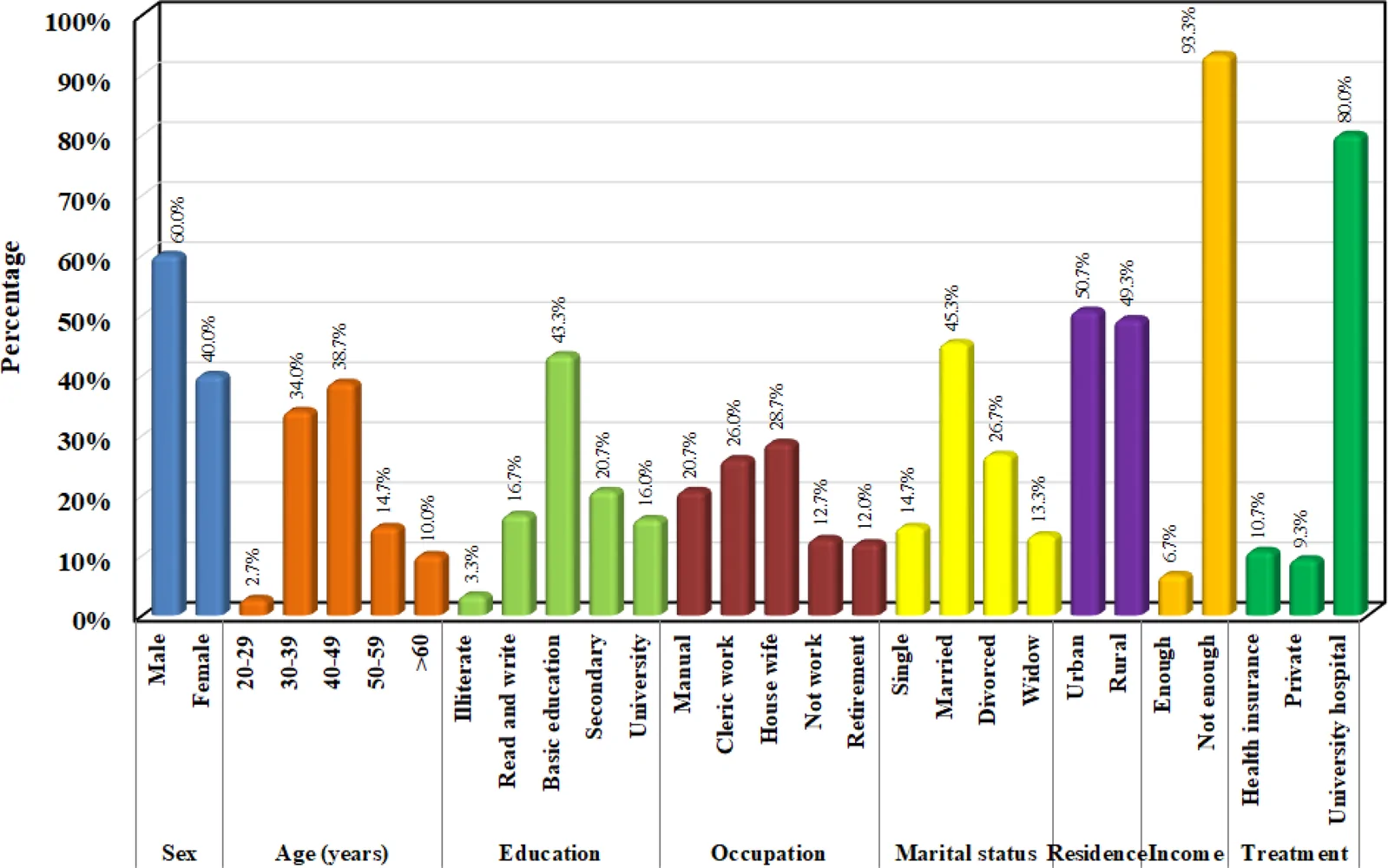
Socio-demographic data (*n* = 150)

Figure 1 Illustrates the socio-demographic characteristics of the study population, highlighting its diversity. The sample includes number of participants are aged 40–49 years 38.7%, while a notable portion is over 60 years 10.0%. Gender distribution shows 60% male and 40% female participants, along with a variety of educational backgrounds and ethnicities. This demographic diversity is essential for understanding how these factors may influence the patient outcomes.

**Fig. 2:**
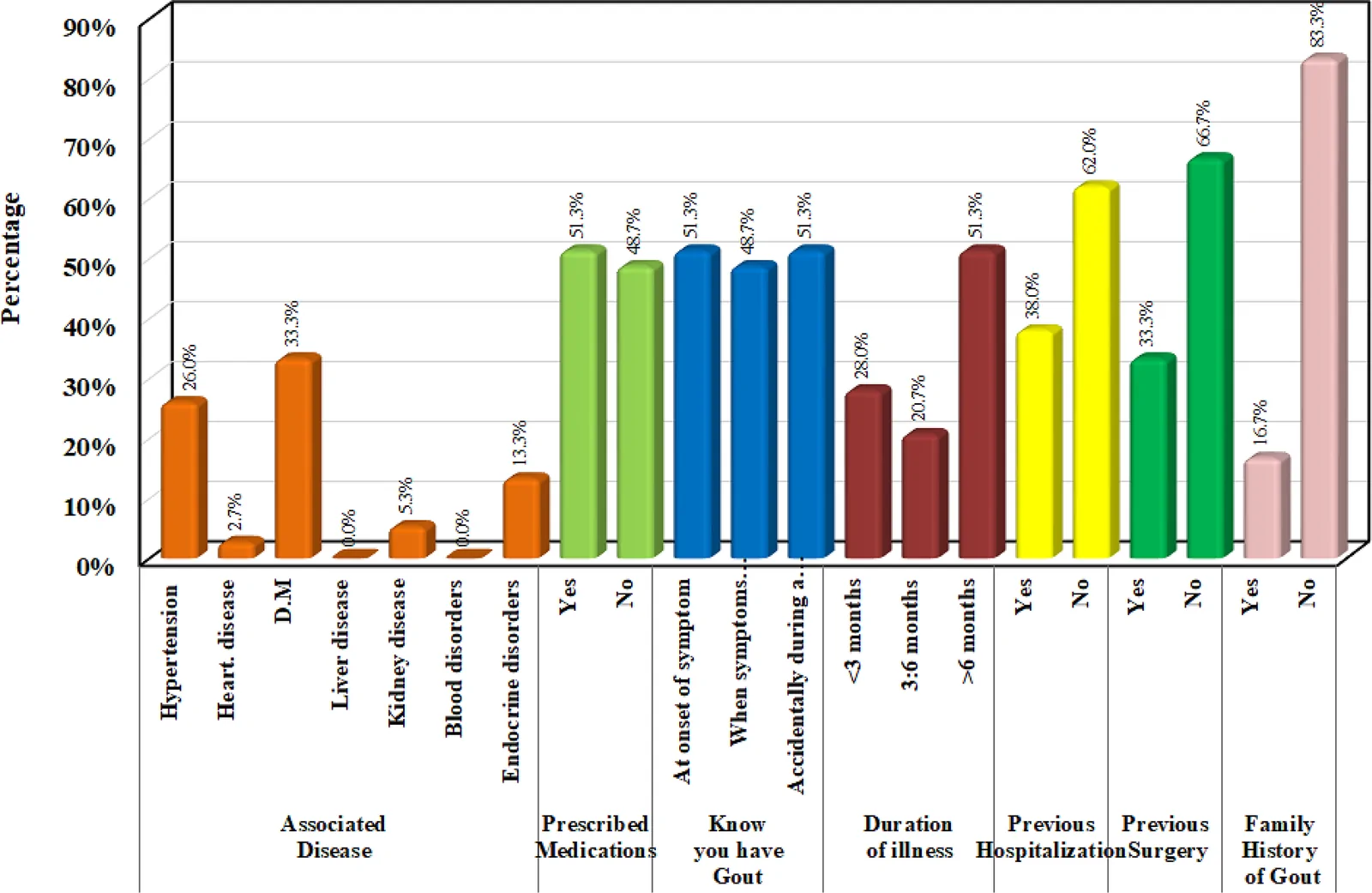
Clinical data (*n* = 150)

Figure 2 Presents the clinical data collected from participants, providing crucial insights into their health status and its implications for medication adherence. Notably, 30% of participants report comorbidities such as diabetes or hypertension, which may complicate adherence efforts. Additionally, 51.3% of participants have a history of previous treatments, emphasizing the need to consider treatment history when examining adherence patterns and potential interventions.

## Discussion

Effective management of gout hinges on patient adherence to treatment, as non-adherence can exacerbate symptoms, increase the likelihood of flare-ups, and result in long-term joint damage. Gout is a chronic condition marked by recurrent acute inflammatory arthritis due to elevated uric acid levels, necessitating ongoing medication to manage these levels and prevent future attacks. However, patients often face several barriers to adherence, including their perceptions of the necessity of treatment, concerns over potential side effects, and challenges in incorporating medication into their daily routines. Recognizing these barriers is essential for developing targeted interventions to enhance adherence and improve patient outcomes [23].

Insights from this research highlight the multifaceted nature of patient adherence, which is heavily influenced by both psychological and socio-demographic factors. Many patient participants reported uncertainty about the clinical importance of their medications and expressed explicit fears regarding potential side effects. Additionally, the research indicates significant correlations between adherence rates and socio-demographic variables such as gender, age, education, and income. By identifying these specific patient-reported barriers, this study emphasizes the necessity of developing targeted clinical interventions. Addressing these patient-centered challenges allows healthcare providers to implement more effective adherence strategies, ultimately leading to better patient outcomes in the long-term management of gout [24].

**The findings of our study illustrated that**,** according to sociodemographic data**, gout is more common in men than in women, which is consistent with previous research. Because estrogen has uricosuric effects in premenopausal women, reducing their risk of hyperuricemia, men often have higher uric acid levels [25]. Most participants were middle-aged, which is by earlier research that suggests gout incidence rises with age due to age-related declines in renal function and prolonged exposure to hyperuricemia [26].

Educational background and occupation are a significant component of gout awareness and management. A large proportion of the participants had only basic education, and many were engaged in clerical or manual labor. Socioeconomic factors influence dietary habits and access to healthcare, both of which are crucial in preventing and managing gout [27]. Additionally, most of the participants reported insufficient income, which could limit their ability to afford medical care and maintain a gout-friendly diet.

**Relative to clinical characteristics and disease awareness**, the study shows a strong association between gout and other chronic conditions, such as diabetes mellitus and hypertension. This supports previous studies linking gout to insulin resistance and metabolic derangements due to shared pathophysiologic mechanisms [28]. In addition, over half of the participants had indicated taking prescribed medication, a marker of the chronic nature of gout and long-term pharmacologic management required.

The participants’ awareness of gout was largely accidental, considering that most of them were discovered to have the disease as part of a general medical checkup, while others saw doctors only when their condition reached alarming proportions. The late diagnosis is concerning since such complications as tophaceous gout and chronic joint damage [5] would have been avoided with an earlier diagnosis. Furthermore, most of the participants had already been diagnosed for years, thus an indication that the disease had progressed to advanced stages before diagnosis.

Hospitalization and Family History, A notable proportion of participants had a history of hospitalization, likely due to severe gout attacks or related complications. Additionally, many had undergone surgery, possibly for joint-related conditions. The study also found that some participants reported a family history of gout, reinforcing the well-established genetic predisposition associated with the disease [29].

Medication adherence is a vital aspect of managing gout, which is chronic inflammatory disease associated with hyperuricemia and the accumulation of monosodium urate crystals in joints [30]. The results from this study uncovered notable challenges to medication adherence and socio-demographic influences on adherence to prescribed gout medications in people with gout.

Looking at the barriers to medication adherence, the study results show that many participants had lack of insight about the need for their medications while a significant portion of participants expressed disbelief in their treatment. The results are consistent with prior research, which highlighted how poor knowledge about the chronicity of gout and the need for urate-lowering therapy contributes to low rates of medication adherence [31]. When asymptomatic, patients often see medications as unnecessary and disengage from their regimen; increasing the chances of recurrent gout flares and incur complications [32].

Concerns about drug safety, adverse drug reactions, and drug dependence were all similar themes in study results. A substantial portion of participants worried that the drug would build up in their bodies, that they would become dependent, or taking the drug would stop the body’s ability to heal on its own. Such beliefs have been identified in previous research, and it highlights the importance of educating patients on safety and the need for long-term urate-lowering therapy [33].

Regarding the Effect of Socio-Demographic Variables on adherence, the study found significant associations between medicine adherence and socio-demographic factors. Males had higher adherence rates compared to females, potentially due to gender differences in health seeking behavior and health beliefs about treatment experiences [34].

Age is also a factor, with the highest adherence rates seen within middle-aged groups. This is consistent with prior studies that indicated youth experience lower adherence rates with chronic diseases due to lifestyle and lower perception of vulnerability to complications [28].

Education was another important factor, with those with a university degree demonstrating higher adherence rates. Previous research suggests that those with a higher degree or education level are much more likely to appreciate the chronicity of gout, and the importance of the ongoing use of medications [35]. However, lower educational levels obviously require support for adherence, which may require further a simplified communication strategy. Housewives, and divorcees had higher adherence, possibly due to the type of daily responsibilities and differences in health prioritization. This supports the concept of making marital and occupational lifestyle factors part of any adherence intervention [5].

Residence and income were also influential; urban residents adhered better than rural residents, likely due to better healthcare access. Furthermore, participants with insufficient income had significantly lower adherence rates. Financial barriers are well-documented obstacles to medication adherence, particularly in chronic conditions requiring lifelong treatment [36]. Policies aimed at improving affordability and access to gout medications could help mitigate these challenges.

Adherence to medication remains one of the most important aspects of effective gout management because it impacts both the disease’s progression as well as the patient’s quality of life. This study’s results suggest important factors for non-adherence, which include skepticism about the need for medication, fear of adverse effects, and the general inconvenience of treatment schedules. Such factors are consistent with previous studies which show that non-adherence to chronic conditions stems from a complex interplay of psychological, social, and organizational issues [23].

An important concern is the patients’ own belief that it is their responsibility to independently determine whether they should go through treatment for the disease. This view shows indication of not fully understanding how chronic gout is as a condition, and that it requires constant medication to manage the disease flares and complications. Other studies indicate that educational techniques can enhance medication adherence by improving patient comprehension of their condition, and the need for medication for them to benefit from it [37].

Another major barrier involved concerns around side effects. Many of the participants expressed discomfort with the amount of chemicals in their bodies and the possible long-term effects of medications. This is reflected in other studies which report that fear of side effects significantly decreases adherence [38]. By addressing these apprehensions through both patient education and shared decision-making approaches, patients may have their fears alleviated, leading to improved adherence.

Psychological barriers, including fear of becoming dependent on medications, also contributed to non-adherence. This is consistent with previous research that has shown that patients with chronic illnesses find the emotional burden of long-term therapy frustrating, leading them to prematurely discontinue therapy [39]. Interventions tailored to patients’ and families’ fears are more likely to be effective in improving adherence, such as cognitive therapy interventions.

Adherence levels were also influenced by socio-demographic factors. Adherence was greater in males than females, while the middle-aged group showed the highest adherence. Education level was also a strong predictor of adherence, being higher in university-educated individuals. And this accords with studies suggestive of a link between higher health literacy and better treatment, importance comprehension and adherence [40].

Adherence was influenced by occupational status and income level, with housewives and those with good income exhibiting better adherence than clerical workers and those with insufficient financial means. The latter [41] is well-documented in prior literature on financial barriers to adherence, where both cost of medication and economic instability are associated with decreased adherence. Policy interventions, such as subsidized medication programs or financial assistance, may help address these issues.

As well as adherence rates were significantly impacted by clinical factors, such as disease duration and mode of diagnosis. Those diagnosed at the onset of symptoms fell in line better than those who sought treatment only when symptoms became severe. Such findings underscore the need for early diagnosis and education of the patient to ensure adherence and sustained adherence. There is a parallel observation in studies on early medical engagement in chronic disease management [42].

Multivariate analysis identified key predictors of adherence, including age, marital status, financial sufficiency, and the act of taking prescribed medications. Notably, individuals who discovered their condition accidentally during routine check-ups were less likely to adhere, possibly due to a lack of perceived urgency in managing their illness. This finding aligns with prior studies indicating that patient perception of disease severity strongly influences adherence behaviors [43].

Such factors are consistent with previous studies which show that non-adherence to chronic conditions stems from a complex interplay of psychological and socio-economic barriers. Building on this, the present study distinguishes itself by grounding these adherence barriers within the specific context of nursing-led clinical insights. While our findings align with established data regarding the prevalence of gout in middle-aged males (Doherty et al., 2018), they uniquely highlight a critical ‘Educational-Perception Gap’ identified by nursing observations. Specifically, the ‘fear of side effects’ found in our results suggests that current physician-led consultations may not be sufficiently addressing the psychological barriers to chronic therapy. Consequently, the practical implication is the urgent need for a nurse-led intervention model, where nurses utilize simplified communication to bridge the literacy gap and manage the ‘flare paradox’ (Rasmussen et al., 2024). By addressing these specific clinical barriers, this research adds to the body of knowledge by reframing gout care as a proactive nursing responsibility rather than reactive episodic treatment [44, 45].

The implications of these findings suggest that targeted interventions are necessary to improve adherence in gout management. Healthcare providers should focus on enhancing patient education, addressing concerns regarding medication safety, and implementing strategies that cater to socio-economic disparities. Additionally, patient-centered approaches, such as motivational interviewing and personalized adherence plans, may be effective in mitigating psychological barriers and fostering sustained treatment engagement.

### Limitations and recommendations

This study presents several key limitations. Firstly, the data collection methods, particularly the use of self-reported measures such as the INAS, may introduce social desirability bias, potentially inflating adherence reports. Additionally, reliance on surveys restricts the depth of understanding regarding patient experiences, which could be more effectively captured through qualitative insights. Secondly, participant selection may impact on the generalizability of the findings; if the sample is not representative of the broader gout patient population, the results may not accurately reflect all patients’ experiences. Notably, most studies were conducted at a single hospital, further limiting generalizability. Furthermore, the outcome measures may not encompass all relevant aspects of treatment adherence and patient outcomes, as factors such as patient satisfaction and quality of life were not thoroughly examined. Lastly, while the data analysis methods employed are appropriate, they may overlook complex interactions among socio-demographic factors and adherence. Acknowledging these limitations is crucial for contextualizing the findings and guiding future research in gout management.

## Conclusions

This study highlights the critical socio-demographic and clinical characteristics of patients with gout, revealing a notably higher prevalence among middle-aged males and a strong association with metabolic disorders. These findings underscore the urgent need for enhanced public awareness campaigns and early screening programs to facilitate timely diagnosis and intervention, ultimately reducing the burden of gout. Furthermore, the observed socioeconomic disparities in disease management highlight the necessity for targeted interventions aimed at improving healthcare access for underserved populations. Promoting lifestyle modifications tailored to these groups is essential for effective gout management. Our findings also reinforce the multifaceted nature of medication adherence in gout management. Psychological barriers, concerns about medication safety, and socio-demographic factors significantly influence adherence rates. To address these challenges, it is critical to implement comprehensive patient education initiatives, simplify medication regimens, and improve healthcare accessibility. We advocate for future research to focus on developing and rigorously evaluating targeted interventions designed to optimize long-term medication adherence among patients with gout. By doing so, we can significantly enhance patient outcomes and improve their overall quality of life. Ultimately, this study contributes to a deeper understanding of the complexities of gout and emphasizes the importance of a holistic approach in both clinical practice and public health strategies.

### Implications of the study

This study on gout treatment adherence identifies critical barriers to patient adherence, including medication costs, forgetfulness, side-effect concerns, and health beliefs. It emphasizes the necessity for tailored nursing interventions, such as simplified dosing, reminder systems, and patient education, alongside multidisciplinary collaboration with pharmacists and social workers to enhance holistic care. By integrating adherence screening into routine practice and advocating for policies to reduce drug costs, the research aims to improve long-term gout management, prevent complications, and alleviate healthcare burdens. Ultimately, it empowers nurses to implement patient-centered strategies that enhance outcomes and quality of life for individuals with gout.

## Data Availability

The datasets used and/or analysed during the current study are available from the corresponding author on reasonable request.

## Abbreviations

MSU: Monosodium Urate
DECT: Dual- energy CT
ACR: American College of Rheumatology
MRI: Magnetic Resonance Imaging
CT: Computed Tomography
NSAIDs: Non-Steroid Anti-Inflammatory Drugs
INAS: Intentional Non-Adherence Scale
UHI: Universal health insurance
UHC: Universal health coverage
SPSS version 25: The Statistical Package for the Social Sciences
